# Heavy ion mutagenesis combined with triclosan screening provides a new strategy for improving the arachidonic acid yield in *Mortierella alpina*

**DOI:** 10.1186/s12896-018-0437-y

**Published:** 2018-05-02

**Authors:** Huidan Zhang, Dong Lu, Xin Li, Yingang Feng, Qiu Cui, Xiaojin Song

**Affiliations:** 1 Shandong Provincial Key Laboratory of Energy Genetics, Qingdao Institute of Bioenergy and Bioprocess Technology, Chinese Academy of Sciences, Qingdao, 266101 Shandong China; 2 Institute of Modern Physics, Chinese Academy of Sciences, Lanzhou, 730000 Gansu China; 3 Key Laboratory of Biofuels, Qingdao Institute of Bioenergy and Bioprocess Technology, Chinese Academy of Sciences, Qingdao, 266101 Shandong China; 4 Qingdao Engineering Laboratory of Single Cell Oil, Qingdao, 266101 Shandong China; 5 University of Chinese Academy of Sciences, Beijing, 100049 China

**Keywords:** Arachidonic acid, Desaturase, Heavy ion mutagenesis, *Mortierella alpina*, Octyl gallate, Triclosan

## Abstract

**Background:**

Arachidonic acid (ARA), which is a ω-6 polyunsaturated fatty acid, has a wide range of biological activities and is an essential component of cellular membranes in some human tissues. *Mortierella alpina* is the best strain for industrial production of ARA. To increase its yield of arachidonic acid, heavy ion beam irradiation mutagenesis of *Mortierella alpina* was carried out in combination with triclosan and octyl gallate treatment.

**Results:**

The obtained mutant strain F-23 ultimately achieved an ARA yield of 5.26 g L^− 1^, which is 3.24 times higher than that of the wild-type strain. In addition, quantitative real-time PCR confirmed that the expression levels of fatty acid synthase (FAS), Δ5-desaturase, Δ6-desaturase, and Δ9-desaturase were all significantly up-regulated in the mutant F-23 strain, especially Δ6- and Δ9-desaturase, which were up-regulated 3- and 2-fold, respectively.

**Conclusions:**

This study confirmed a feasible mutagenesis breeding strategy for improving ARA production and provided a mutant of *Mortierella alpina* with high ARA yield.

## Background

Arachidonic acid (ARA), which is a ω-6 polyunsaturated fatty acid, has a wide range of biological activities as it is a prerequisite for the synthesis of prostaglandins, thromboxane and leukotrienes [1]. For a long time, ARA was mainly obtained from animal tissues, such as animal livers, fish oil, and pig adrenal glands, which have low ARA contents and are limited sources [2]. Therefore, major efforts have been made to identify alternative sources of ARA. *Mortierella alpina*, which can accumulate a large amount of ARA in a short growth cycle, has received wide attention [3] and is considered the most prominent ARA-rich oil producer [4, 5]. Nevertheless, low yield and high cost are still the bottleneck for large-scale production of ARA by *M.alpina.*

At present, the genetic engineering method has been widely used in the increase of ARA production of *M. alpina* [6–8]. However, the metabolic regulation network of *M. alpina* is very complex [9, 10], changes in one or two genes are not easy to produce a transformant with the desired trait. Therefore, the mutagenesis method is still one of the effective methods to obtain high yield strains. In general, mutagenesis methods can be divided into two categories, physical mutagenesis and chemical mutagenesis. Heavy ion mutagenesis is a new physical mutagenesis technique with high bioavailability, high energy density, poor repair effects and good spatial resolution of energy deposition compared with traditional radiation sources (e.g., UV, γ- and χ-rays); thus, it can produce more extensive mutation [11–13]. As a chemical mutagenesis method, 5-fluorouracil (5-FU) is a structural analog of uracil that inhibits the synthesis of DNA and some RNA [14, 15], and it has been successfully used for microbial mutagenesis and cancer treatment as an effective chemical mutagen [16, 17]*.*

**Fig. 1:**
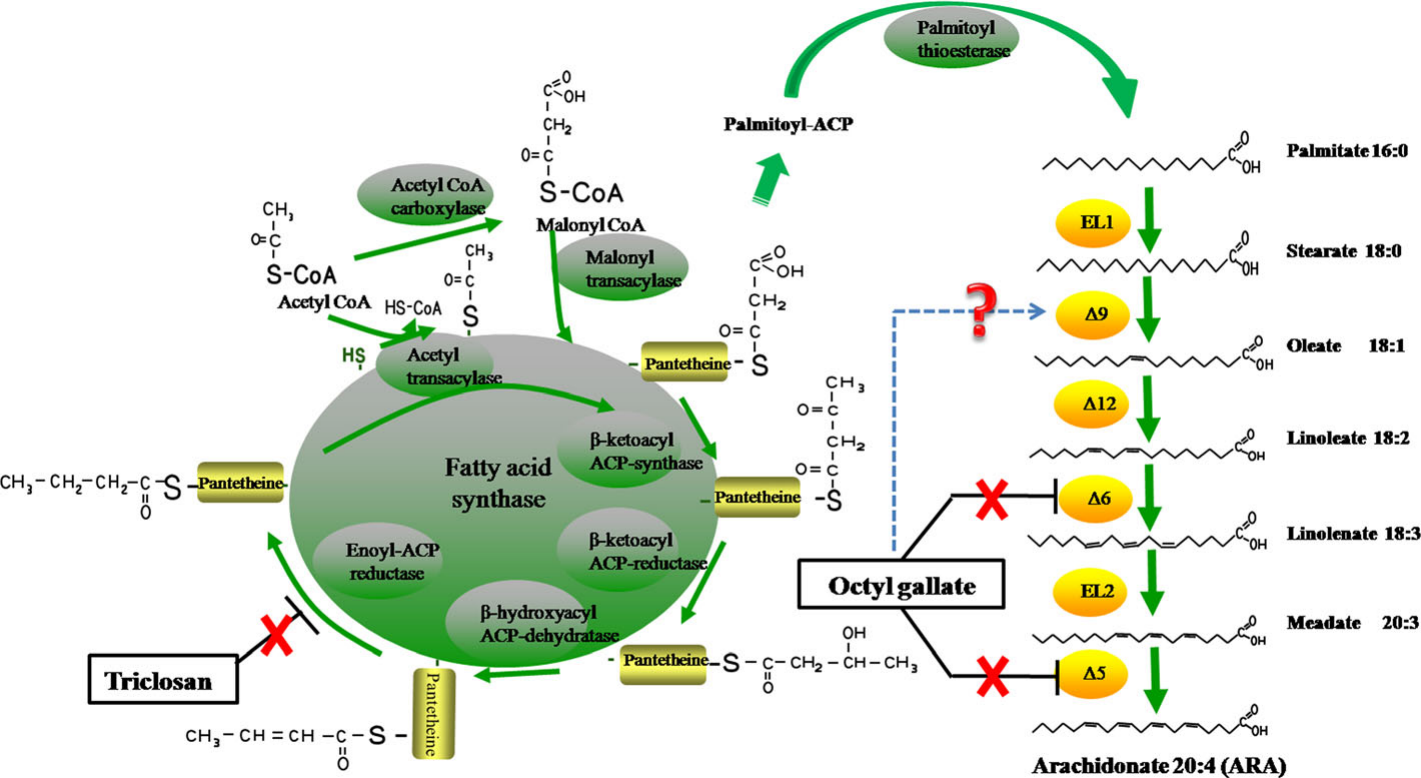
The ARA biosynthesis pathway and the action sites of inhibitors in *Mortierella alpina*

Quickly identifying and screening out high-yield mutants are also important issues for breeding research. Directional screening is more effective than random selection and can reduce the amount of work [13]. From the anabolism pathway of ARA (Fig. 1), two key regulation points have been identified. The first is the synthesis of palmitic acid by fatty acid synthase (FAS), and the other is a series of desaturation reactions with stearic acid as the substrate that are catalyzed by Δ9 and Δ6- desaturase [18, 19]. Triclosan has a broad-spectrum antibacterial effect that can inhibit the activity of FAS by inhibiting the *fabI* locus [20]*.* Mutants that grow normally on a certain concentration of triclosan plates can be identified as having relatively high FAS activity, making them good candidates for lipid production. In addition, octyl gallate is a type of antioxidant that strongly inhibits the activities of fatty acid desaturases in fungi and viruses [21–23]. The octyl gallate mechanism of action may also involve membrane interactions that lead to the loss of membrane potential and cell gap leakage. Ken-ichi Fujita observed this phenomenon when studying the inhibitory effects of octyl gallate on *Zygosaccharomyces bailii* and *Saccharomyces cerevisiae* [24, 25]. Therefore, octyl gallate can be used as an effective reagent for screening high-yield ARA strains of *M. alpina.*

In this study, the wild-type strain SD003 encountered two rounds of mutagenesis and targeted screening. First, heavy ion mutagenesis was combined with triclosan to screen strains with high FAS activity. Second, strains with high desaturase activity were screened by using octyl gallate after mutagenized with 5-fluorouracil. The two-step targeted screening strategy greatly improved the screening efficiency compared with the previous method of random selection. We also investigated the mechanism by which the ARA yield is increased in the mutagenized strain as well as the batch fermentation conditions for optimal production.

## Methods

### Strains and culture conditions

In the present study, *M. alpina* SD003 (CGMCC No.7960) was used as the initial strain. GY medium (glucose, 20 g L^− 1^; yeast extract, 10 g L^− 1^; and agar,20 g L^− 1^) was used to screen *M. alpina*. The seed culture medium contained glucose, 30 g L^− 1^; yeast extract, 6 g L^− 1^; KH_2_PO_4_, 3 g L^− 1^; NaNO_3_, 2.8 g L^− 1^; and MgSO_4_•7H_2_O, 0.5 g L^− 1^. The medium for the shake-flask and bioreactor fermentation cultures contained glucose, 80 g L^− 1^; yeast extract, 11 g L^− 1^; KH_2_PO_4_, 3.8 g L^− 1^; NaNO_3_, 3.5 g L^− 1^; and MgSO_4_•7H_2_O, 0.5 g L^− 1^. The seed cultures were grown at 25 °C with shaking at 200 rpm for 2 d and were then inoculated into shake flasks at 10% (*v*/v) inoculum and cultured at 25 °C with shaking at 200 rpm for 7 d. The bioreactor cultivations were performed in 5-L Biostat B plus bioreactors (Sartorius Stedim Biotech, Germany) containing 3 L of medium at 25 °C with stirring at 200 rpm for 10 d. Triclosan, octyl gallate, and 5-fluorouracil were purchased from Sangon Biotech Shanghai Co., Ltd. (Shanghai, China), Shanghai Yuanye Biological Technology Co., Ltd. and Alfa Aesar (China) Chemical Co., Ltd., respectively.

### Heavy ion beam irradiation and triclosan screening

**Fig. 2:**
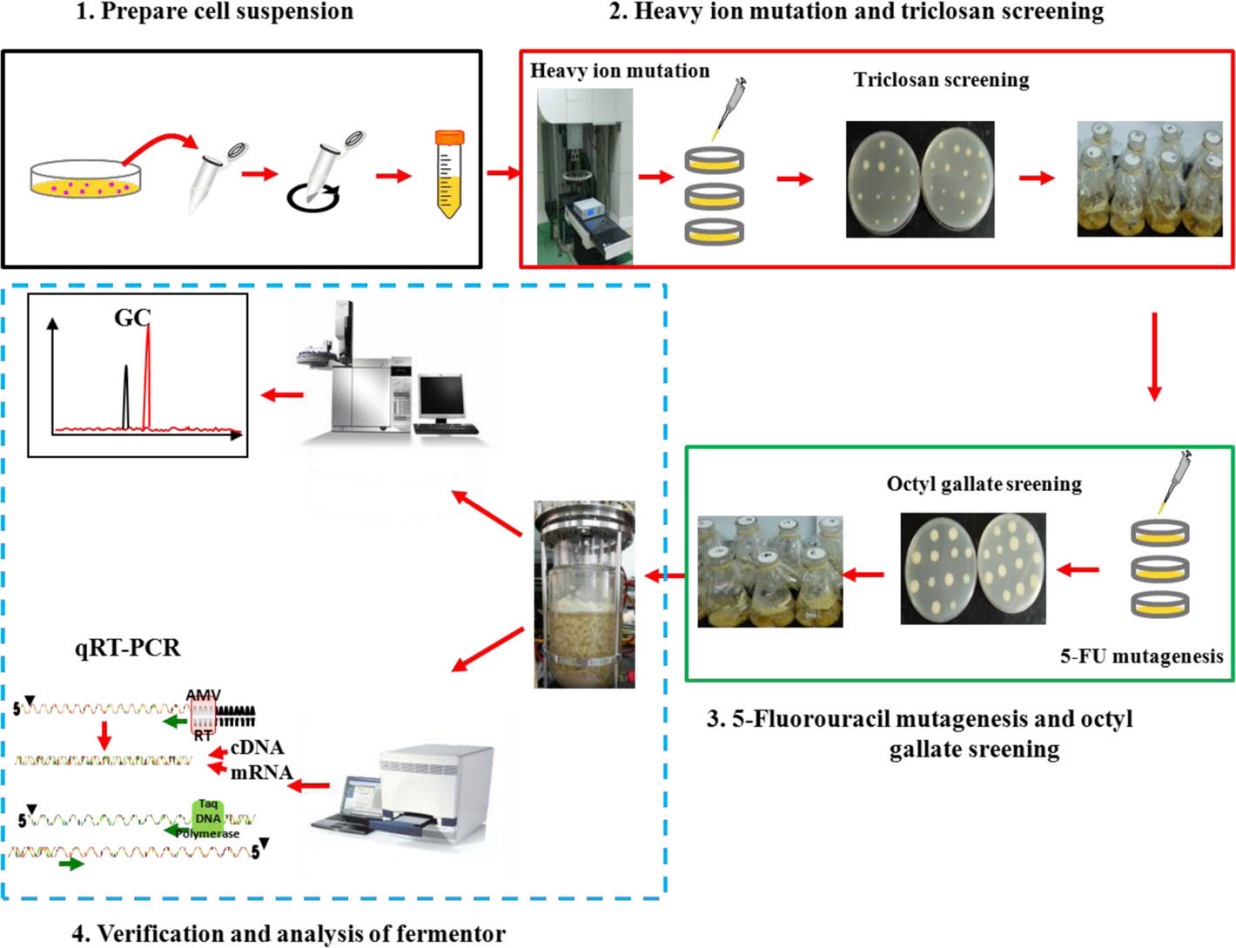
Schematics of the mutant screening experiment

To obtain a stable, high-yield ARA strain, the overall experiment included a four-step screening program with gradually increased screening efficiency, as shown in Fig. 2. The detailed process is described below.

The first step was the preparation of the cell suspension. Spores of *M. alpina* SD003 were rinsed with sterile water and filtered through three layers of sterile lens paper. The spore suspension was centrifuged at 1900×g for 10 min and diluted with sterile water to a concentration of 10^6^ spores mL^− 1^.

Then, the heavy ion beam irradiation experiments were carried out at the Heavy Ion Research Facility in Lanzhou (HIRFL) at the Institute of Modern Physics at the Chinese Academy of Sciences. A ^12^C^6+^ heavy ion beam was used with an energy of 80 MeV μ^− 1^ and an LET (the energy transferred per unit length) of 31 keV μm^− 1^. The heavy ion irradiation dose was 0, 40, 80, 120, 180, or 240 Gy. The irradiated spore suspension was diluted to 10^3^ and 10^4^ spores mL^− 1^, and 100 μL of the spore suspensions were coated on GY plate culture medium. Each dilution was performed in triplicate. After incubation in the dark at 25 °C for 3d, the mortality rate was calculated using the unirradiated spore suspension as a control.

To determine the optimal antimicrobial concentration of triclosan, the unirradiated spore suspension was treated with different triclosan concentrations (0, 0.1, 0.5, 0.6, 0.8, 1.0, 1.5, 2, 2.5 and 3 mgL^− 1^) on GY plates at 25 °C for 3 d, and the mortality rate was calculated. After determining the optimum concentration, the irradiated spores were properly diluted and incubated on plates containing this concentration of triclosan. After the passage of three generations, stable mutants were cultured and tested for ARA production. Consequently, the strain with the highest ARA yield, mutant Z-44, was selected for the next round of 5-fluorouracil mutagenesis.

### 5-fluorouracil mutagenesis and octyl gallate screening

5-fluorouracil mutagenesis was carried out as the third step of the breeding experiment. The mutant Z-44 obtained from the preceding steps was cultured in PDA medium for 10 d and then washed with 0.9% NaCl to prepare a spore suspension. One milliliter of the spore suspension (10^8^ cells) was inoculated into a 50-mL Erlenmeyer flask containing 10 mL of 0.9% NaCl. After incubation for 12 h at 25 °C with shaking at 120 rpm, 5-fluorouracil was added to this starvation medium at a final concentration of 20 μg mL^− 1^. The mixture was incubated in a rotary shaker for 5 h, 10 h and 36 h at 25 °C with shaking at 120 rpm.

After 5-fluorouracil mutagenesis, the mutated spores were spread onto GY plates containing 14 μg mL^− 1^ of octyl gallate.

### Determination of the genetic stability of the mutant

Mutant strains were passaged for 10 generations on PDA slants. Changes in the ARA production performance indexes of the strains were analyzed before and after passage.

### Analytical methods

#### Determination of cell dry weight

Fermentation broth was filtered at room temperature, and the mycelia were washed twice with distilled water. The cell pellets were dried to a constant weight at 50 °C.

#### Total lipid extraction and fatty acid determination

Lipid extraction and transesterification were carried out according to a previously described method [26]. Total lipids were extracted using approximately 100 mg of mycelia (dry weight). Fatty acid methyl esters (FAMEs) were obtained by reacting the lipids with 2% sulfuric acid in methanol (*v*/v) at 85 °C for 2.5 h. FAMEs were extracted with n-hexane and analyzed using a gas chromatograph (Agilent Technologies) equipped with an HP-INNOWAX (30 m × 0.25 mm, 0.25 μm film thickness) capillary column. The oven temperature was set to 100 °C for 1 min and was then raised to 250 °C at a rate of 15 °C per min before being allowed to stand at 250 °C for 5 min. Peak detection was performed using a flame ionization detector (FID). The carrier gas was nitrogen, and the split ratio was 1:19. The injection volume was 1 μL.

#### Quantitative real-time PCR (qRT-PCR) analysis

After 7 d of fermentation, total RNA was extracted with an RNeasy Plant Mini Kit (QIAGEN, Germany) according to the manufacturer’s protocol. cDNA was prepared using a Revert Aid First Strand cDNA Synthesis Kit (Thermo Scientific), and the FastStart Universal SYBR Green Master Mix (ROX) was used for the PCR reactions. The qRT-PCR primers are listed in Table 1. Differences in gene expression were detected using a LightCycler R480 Real-time Detection System (Roche). 18SRNA was used as an internal control to normalize the expression levels. The comparison of CT method (2^-ΔΔCT^) was used to analyze the gene expression differences.

**Table 1:** Primers used in this study

Primers	Sequence(5′-3′)	Description
FAS-RTF	TCCTTTCTATGGTGCTTCCTCA	
FAS-RTR	ATCTTACCTTGCTGCCGTTCT	FAS RT-qPCR
Δ5-RTF	TGTCTGGAAGATTCTGGGAGC	
Δ5-RTR	TTTGGTTGGGCTTGATACGAC	Δ5 RT-qPCR
Δ6-RTF	TGTTCTGGCAGCAGTGCGGATGG	
Δ6-RTR	GGCGTGGTGAGTGTTGTGCTTGTCC	Δ6 RT-qPCR
Δ9-RTF	TCGTTCAGTGGCAGCACAAGA	
Δ9-RTR	CAAGACGGAGGATAGCAGCATA	Δ9 RT-qPCR
Δ12-RTF	TGGGTGCTGGCTCACGAGTGT	
Δ12-RTR	CCAGTGGCCTTGTGGTGCTTC	Δ12 RT-qPCR
18S-RTF	CGTACTACCGATTGAATGGCTTAG	
18S-RTR	CCTACGGAAACCTTGTTACGACT	Internal control for RT-qPCR

## Results

### Heavy ion beam irradiation-induced *M. alpina* mortality

**Fig. 3:**
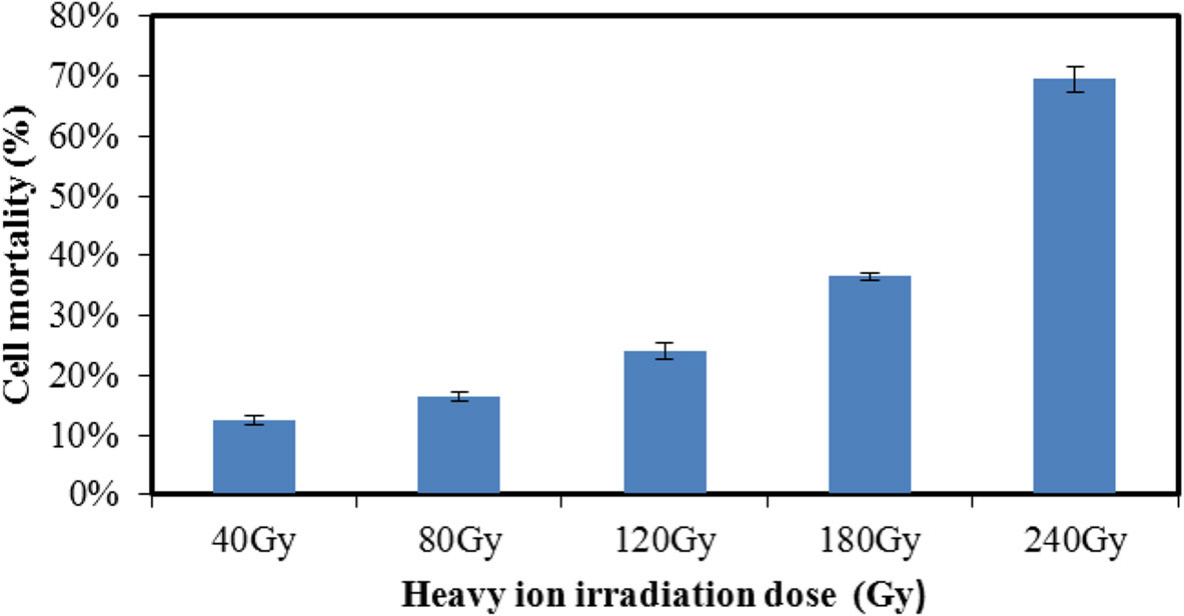
Mortality of *Mortierella alpina* SD003 treated with different doses of heavy ion beam irradiation. Data are the means of three replicate experiments, and the error bars indicate standard deviation

Heavy ion mutagenesis has been successfully applied to plants [27, 28] and some industrial microbial mutagenesis breeding programs [11, 29], but as far as we know, this study is the first to use it with *M. alpina*. Due to a lack of reference conditions for the heavy ion mutagenesis of *M. alpina*, we investigated the lethality of five doses of heavy ion beam irradiation in this experiment. As shown in Fig. 3, at 40, 80, 120, 180 and 240 Gy, the lethality rates were 13%, 17%, 25%, 37%, and 71%, respectively. The lethality of spores increased as the dose increased, with a sharp increase to 71% at 240 Gy. In general, a lethality rate of 70–80% yields a high positive mutation rate [30]. Therefore, the heavy ion beam irradiation mutagenesis was performed at a 240 Gy dose in this study.

### Screening of high-yield ARA mutants with triclosan

Determining how to screen high-yield strains efficiently from a large number of mutants is the key issue in mutagenesis breeding experiments. When traditional shake flask fermentations are used as the means of screening, the screening cycle is long and troublesome. However, when triclosan is used as the screening method, the screening scope is much narrower, and the screening efficiency is greatly improved.

To determine the susceptibility of the wild-type strain to triclosan, *M. alpina* SD003 was spread onto GY plates containing different concentrations of triclosan. Triclosan was found to strongly inhibit the growth of the wild-type strain. The number of colonies decreased with increasing triclosan concentrations. The lethal rate was approximately 92.8% when the triclosan concentration was 2 mg L^− 1^. Thus, 2 mg L^− 1^ of triclosan was selected as a suitable concentration for screening mutants after heavy ion beam irradiation mutagenesis.

**Fig. 4:**
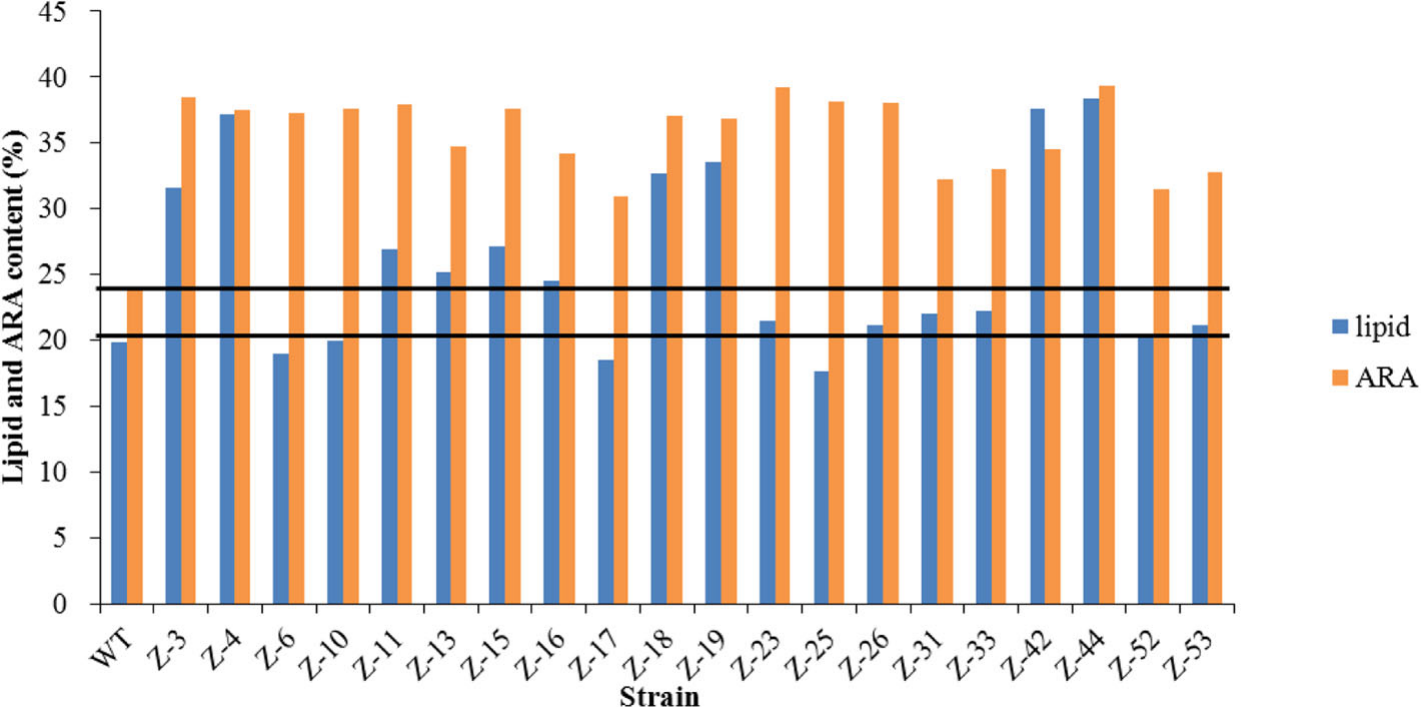
The total lipid and ARA contents of the isolated mutants in 250 mL shake flask after heavy ion beam irradiation mutagenesis. The initial glucose concentration was 80 g L^− 1^. “WT” indicates the wild-type strain. Numbers from Z-3 to Z-53 indicate the mutants

The spore suspensions treated with a heavy ion beam irradiation dose of 240 Gy were properly diluted, spread onto GY plates containing 2 mg L^− 1^ triclosan and incubated at 25 °C for 3 d. Only mutant strains with high FAS activity could grow and form large colonies. We randomly selected 120 large colonies and screened them three times on GY plates containing 2 mg L^− 1^ triclosan. Fifty-three mutants were still able to grow steadily. As shown in Fig. 4, 20 strains with high lipid and ARA contents were further screened via shake flask fermentations. The mutant Z-44 had the highest ARA production, with lipid and ARA yields of 9.65 g L^− 1^ and 3.80 g L^− 1^, which were 1.10 times and 2.06 times higher than that of the wild-type strain, respectively (Table 2).The mutant strain Z-44 mainly had an increased total lipid content (38.3% of the biomass), which was nearly double that of the wild-type strain (19.8%). Analyzing the fatty acid composition (Table 3) revealed that the ARA content of mutant Z-44 was significantly improved (from 27.04% to 39.37%) and that the ratio of C16:0 and C18:1 decreased significantly.

**Table 2:** Productivity of wild-type strain, mutant strain Z-44 and F-23

Strains	Biomass (g L^− 1^)	Lipid content (%)	Lipid yield (g L^− 1^)	ARA content (%)	ARA yield (g L^− 1^)
WT	23.24 ± 0.46	19.8 ± 0.36	4.60 ± 0.56	27.04 ± 0.63	1.24 ± 0.18
Z-44	25.2 ± 0.93	38.3 ± 0.97	9.65 ± 0.26	39.37 ± 0.28	3.80 ± 0.65
F-23	28.2 ± 0.36	38.0 ± 0.25	10.72 ± 0.41	49.08 ± 0.17	5.26 ± 0.48

**Table 3:** Analysis of fatty acid composition of wild-type strain and mutant strains

Strains	Fatty acids content (% total fatty acids)							
	C14:0	C16:0	C18:0	C18:1	C18:2	C18:3	C20:3	C20:4
WT	2.83 ± 0.07	18.33 ± 0.46	13.32 ± 0.99	25.65 ± 1.79	5.72 ± 0.52	2.88 ± 0.14	3.75 ± 0.12	23.99 ± 1.92
Z-44	1.13 ± 0.04	11.26 ± 0.93	22.35 ± 1.37	7.91 ± 0.27	6.45 ± 0.16	3.83 ± 0.05	2.64 ± 0.09	39.37 ± 0.28
F-23	0.61 ± 0.01	10.93 ± 0.02	13.95 ± 0.01	8.48 ± 0.29	5.35 ± 0.12	3.97 ± 0.07	3.53 ± 0.02	49.12 ± 0.06

Data are expressed as means ± standard deviations

### 5-fluorouracil mutagenesis and octyl gallate screening of strains

To further improve the yield of ARA, the mutant strain Z-44 was used as the starting strain for the second round of mutagenesis with 5-fluorouracil, which is a compound similar to uracil that can be converted into effective fluorouracil deoxynucleotides that interfere with the synthesis of DNA. First, we studied the lethality of 5-fluorouracil after different treatment times. The results showed that the lethality rate was 50% at 5 h, 71% at 10 h and 93% at 36 h for 20 μg mL^− 1^ 5-fluorouracil treatment. Therefore, considering the operability of the experiment, we ultimately selected 10 h as the processing time.

Alkyl gallate salts have been reported to be antioxidants with strong inhibitory effects on the Δ6-fatty acid desaturase in the ARA-producing fungi *M. alpina* 1S-4, and the inhibition of octyl gallate was the most obvious [25]. Therefore, strains resistant to octyl gallate may have high fatty acid desaturase activities, thereby promoting the formation of ARA. The lethal rate of octyl gallate at 12 μg mL^− 1^ was 50%, and it was 100% at 25 μg mL^− 1^. The lethal rate approached 86% when the spores were spread on GY plates containing 12 μg mL^− 1^ octyl gallate after treatment with 5-fluorouracil for 10 h. After 3 rounds of screening, 80 strains were selected for shake flask fermentation validation.

**Fig. 5:**
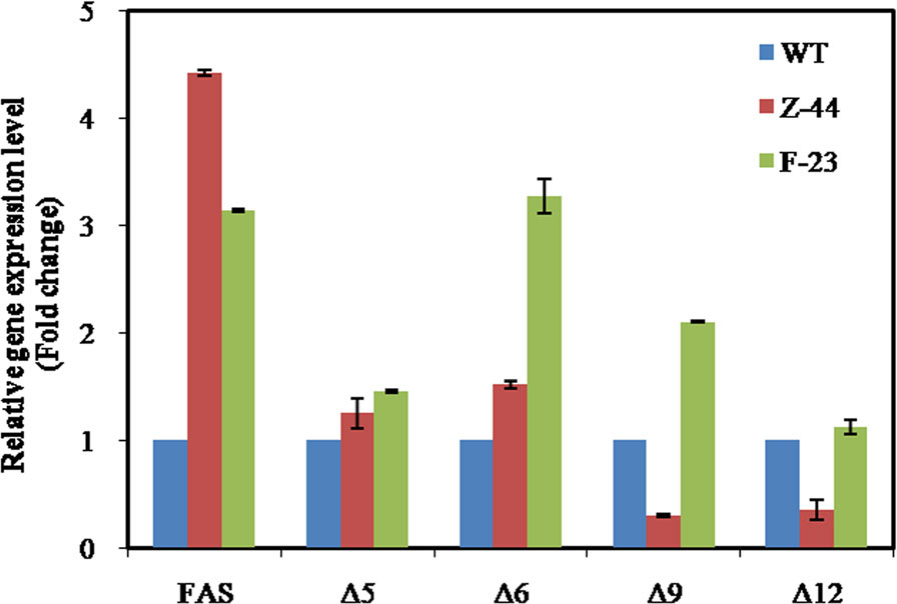
Relative gene expression levels of fatty acid synthase (FAS), Δ5- desaturase, Δ6-desaturase, Δ9- desaturase and Δ12- desaturase of the wild-type, mutant Z-44 and mutant F-23 strains

The ARA yield of the mutant strain F-23 was 5.26 g L^− 1^, which was the highest yield observed in the mutants and 3.24 times higher than that of the wild-type strain (1.24 g L^− 1^). The ARA content of the mutant F-23 was 49.08%, which was 81.51% higher than that of the wild-type strain (27.04%) and 24.66% higher than that of the mutant Z-44. Analysis of the fatty acid composition revealed that the C18:0 ratio was decreased significantly due to the rising catalytic capacity of the Δ9-desaturase (Fig. 5). The growth characteristics of the wild-type and two mutant strains, Z-44 and F-23, are shown in Tables 2 and 3.

### Expression levels of the endogenous FAS and Δ5, Δ6, Δ9 and Δ12-desaturase genes in the wild-type and mutant strains

To confirm the roles of triclosan and octyl gallate in the fatty acid synthesis pathway of *M. alpina*, five genes, fatty acid synthase, Δ5-desaturase, Δ6-desaturase, Δ9-desaturase, and Δ12-desaturase, that are related to ARA synthesis were analyzed by qRT-PCR at the mRNA level. As shown in Fig. 5, the mRNA expression level of FAS in mutant Z-44 was significantly up-regulated at a level 4.4 times higher than that of the wild-type strain. Therefore, the fatty acid synthase was the main locus of action of triclosan; some other studies have also demonstrated that triclosan inhibited the activity of enoyl-ACP reductase, which is a module of the FAS complex [19, 31, 32].

For the mutant F-23, which is a further mutagenized strain of the mutant Z-44, not only the expression level of FAS was increased by 3.1 times but the expression levels of all of the desaturases were also up-regulated, especially the Δ6 and Δ9-desaturases, which were significantly up-regulated by 3.2 and 2.1 times, respectively, compared to those of the wild-type strain (Fig. 5).

### Genetic stability of the mutant

To investigate the genetic stability of the mutagenized strain, strain F-23 was cultured for 10 generations. Fermentation was carried out every five generations, and the biomass, lipid and ARA yields were measured three times. As shown in Table 4, the ARA yield was approximately 5.5 g L^− 1^, and the biomass was stable at approximately 29 g L^− 1^ after 10 generations, indicating that the mutant F-23 had good genetic stability.

**Table 4:** Genetic stability of mutant strain F-23

Passage number	Biomass (g L^−1^)	Lipid content (%)	Lipid yield (g L^− 1^)	ARA content (%)	ARA yield (g L^− 1^)
1	29.20 ± 0.64	38.97 ± 0.26	11.37 ± 0.17	50.36 ± 0.57	5.73 ± 0.15
5	28.52 ± 0.39	37.06 ± 1.07	10.57 ± 0.45	48.39 ± 0.72	5.12 ± 0.29
10	28.93 ± 0.83	38.25 ± 0.35	11.06 ± 0.22	49.12 ± 0.06	5.43 ± 0.11

### Batch fermentation of mutant F-23

**Fig. 6:**
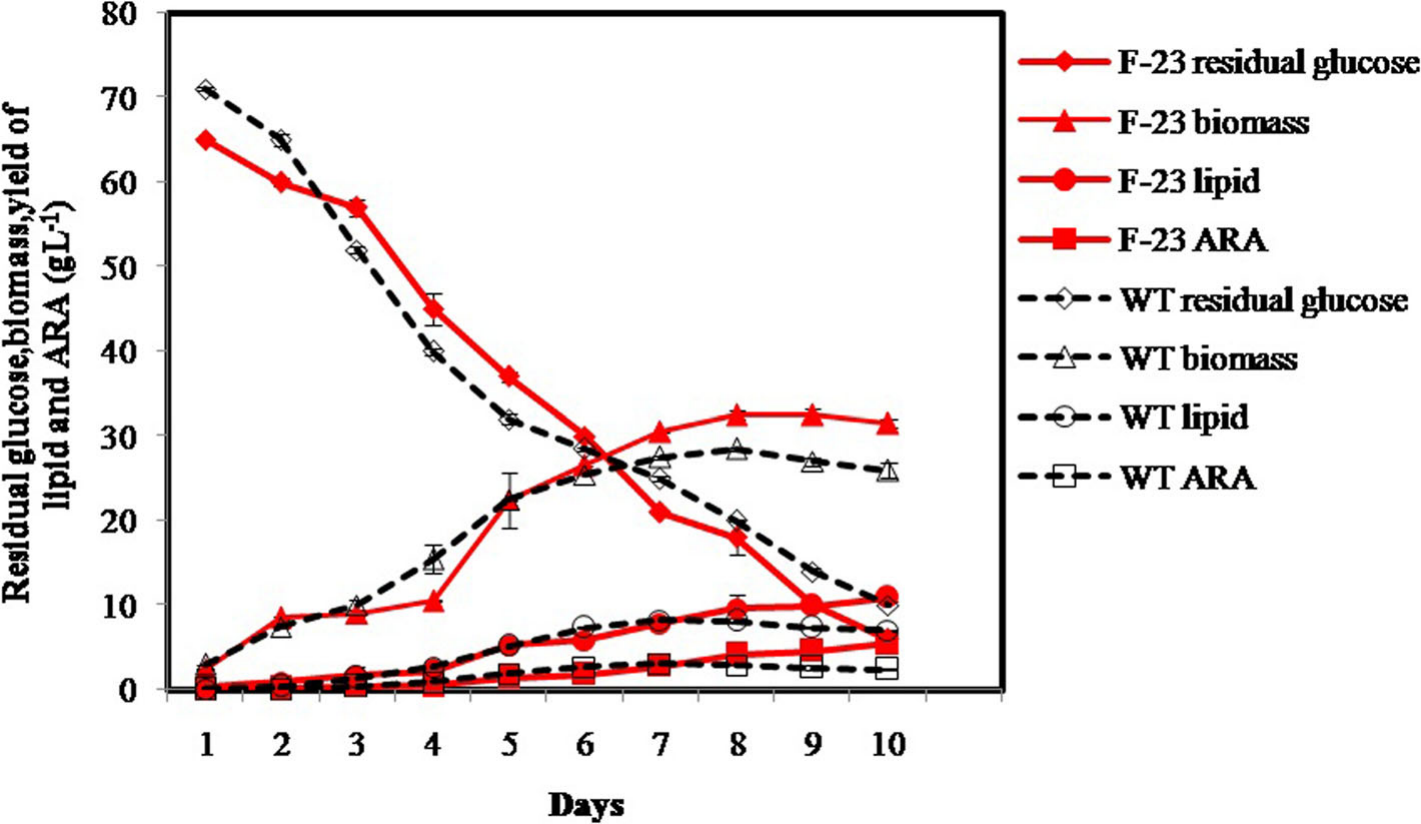
Growth curves of the mutant F-23 and wild-type strains in 5-L fermenters. 80 g L^− 1^ glucose was supplemented to the medium once a day from the first day. Red represents the mutant F-23 strain, and black represents the wild-type strain

The mutant F-23 was further evaluated in batch fermentations in a 5-L bioreactor. The growth curves of F-23 and wild-type SD003 are shown in Fig. 6. The growth of the mycelia can be seen to experience three typical periods: delay, rapid growth and decay. The mutant F-23 had a relatively longer delay period (approximately 4 d) than wild-type SD003, possibly due to effects of mutagenesis on the expression of some genes or enzymes in vivo, resulting in a growth delay. The glucose consumption rate of both strains was basically the same. Subsequently, a more efficient logarithmic phase was observed in mutant F-23, which had a final biomass of 32.5 g L^− 1^, 14.04% higher than strain SD003. Finally, the decay period was observed in both of these strains after 8 d, and it was more obvious in the wild-type strain.

The total lipid and ARA yields almost synchronously accumulated with mycelial growth. After 10 d of fermentation, the lipid yield of mutant F-23 reached a maximum of 10.93 g L^− 1^, 1.55 times of that of wild-type strain SD003 (7.06 g L^− 1^). Meanwhile, the ARA yield of mutant F-23 was 5.46 g L^− 1^, 2.22 times of that of strain SD003 (2.45 g L^− 1^). These results indicate that mutant F-23 could be a valuable candidate strain for producing ARA.

## Discussion

Due to the lack of markers and low transformation frequencies, there are still some technical problems in the simultaneous transformation of several genes of *M. alpina* by molecular methods. The mutagenesis breeding method is still an effective breeding method, because it could change many characteristics without requiring much biochemical and genetic information of the strain. In this study, heavy ion beam irradiation mutagenesis combined with directional screening was carried out and greatly improved the work efficiency. This is the first report about applying heavy-ion irradiation mutagenesis on *M. alpina* breeding.

So far, researchers have carried out a variety of ways to mutagenize on *M. alpina*, such as UV, γ- and χ-rays, etc. [33]. Compared with the traditional physical mutagenesis (e.g., UV, γ- and χ-rays), heavy ion beam irradiation mutagenesis has higher LET **(**the energy transferred per unit length) that can induce more biological damage than other types of radiation, which is difficult to repair. Therefore, mutations caused by heavy ion mutagenesis are relatively stable [34]*.* The LET value of the heavy beam used in this study is 31 keV μm^− 1^, while the LET values of the χ-ray and γ-ray are only 0.2 and 2.0 keV μm^− 1^ respectively [35]. On the other hand, the heavy ion ionization peak (Bragg peak) is sharper. Thus, the interaction area between the heavy ion beam and the biological sample is more concentrated, which results in a higher mutation rate [36]. Therefore, heavy ion mutagenesis can be used for *M. alpina* as a more efficient breeding method.

Usually, a lot of works are needed during the mutants screening. So, in order to reduce the workload, this study attempts to establish a rapid pre-screening method. Triclosan has been used in some studies on lipid accumulation [12], but whether triclosan could improve ARA production is not clear. From our results, using the triclosan screening method, twenty strains screened from all the 53 mutants had an ARA content increasing by at least 30%, with the highest ARA content of mutant Z-44 increased nearly 40%. The positive mutation rate is 38%, which is higher than other methods and greatly improves the work efficiency. These results indicated that the method using heavy ion mutagenesis combined with triclosan screening was an effective method for improving ARA production of *M. alpina.*

In order to investigate the effect of triclosan on lipid and ARA synthesis, we used qRT-PCR to investigate the mRNA expression levels of desaturase and fatty acid synthase (FAS) in *M. alpina*. The results showed that the mRNA expression level of FAS in mutant Z-44 was significantly increased (4.4 times), indicating that triclosan strongly affected the FAS activity, and the selected mutant had strong FAS activity (Fig. 5). This is also consistent with the fatty acid composition of Z-44 in Table 3. The contents of palmitic acid (C16:0) in mutant Z-44 were significantly decreased, and the proportion of stearic acid (C18:0) in the total fatty acids is still high. This phenomenon may cause by the increase of FAS activity and the low expression levels of the four desaturases, especially the low expression of Δ9-, Δ12-desaturase, which still blocked the ARA biosynthesis (Figs. 1 and 5). Therefore, the enhancement of desaturase expressions or activities were selected as the second round screening strategy.

According to the previous report, octyl gallate can strongly inhibit the activity of Δ5- and Δ6- desaturase of *M. alpina* [37], so we decided to use it as the screening agent. The results of qRT-PCR showed that the mRNA expression levels of all the desaturases of mutant F-23 were improved, especially the expression levels of Δ9- and Δ6-desaturases, which suggested that the main action sites of octyl gallate may be the Δ6- and Δ9- desaturase. These results suggested that octyl gallate can inhibit the activity of not only Δ5- and Δ6-desaturases, but also the other desaturases, especially Δ9-desaturase (Fig. 1). In addition, compared with the Z-44 mutant, the content of C18: 0 and C18: 2 in mutant F-23 were decreased significantly. In particular, the content of 18:0 decreased from 22.35% to 13.95%, with a drop of 37.58%. This may be due to the activity increase of the Δ9-, Δ6- desaturase, which increases the flux of ARA biosynthesis, thereby further increasing the ARA content (from 38.37% to 49.12%).

Our results showed that compared with the wild type, the lipid and ARA yield of mutant F-23 were obviously increased 1.33 and 3.24 times, respectively. The genetic trait of mutant F-23 was stable during 10 passages. Based on our results, the ARA yield will be further improved, if we next carry out the fermentation optimization and the genetic transformation methods, such as the overexpression of VHb, ME and other genes [38–41]. In general, our study provides a good method and strategy for screening microorganisms with high yield of unsaturated fatty acids.

## Conclusions

In conclusions, the improvement of the industrial strain was an effective way to reduce the ARA production costs. In this study, mutant F-23 was selected after heavy ion beam irradiation combined with triclosan and octyl gallate treatment. Compared with the wild-type strain, the total lipid and ARA yields were increased by 1.33 and 3.24 times, respectively. These results provide a good method and strategy for screening microorganisms with high yield of unsaturated fatty acids.

## Abbreviations

5-FU: 5-fluorouracil
ARA: arachidonic acid
CGMCC: China General Microbiological Culture Collection Center
DGLA: dihomo-γ-linolenic acid
FAMEs: fatty acid methyl esters
FAS: fatty acid synthase
FID: flame ionization detector
GLA: γ-linolenic acid
GY: screening medium
HIRFL: Heavy Ion Research Facility in Lanzhou
LA: linoleic acid
LET: the energy transferred per unit length
*M. alpina*: Mortierella alpina
ME: malic acid
PDA: potato dextrose agar medium
VHb: *Vitreoscilla* hemoglobin
